# Sepsis, Management & Advances in Metabolomics

**DOI:** 10.7150/ntno.94071

**Published:** 2024-02-25

**Authors:** Swarnima Pandey

**Affiliations:** University of Maryland, School of Pharmacy, Department of Pharmaceutical Sciences, Baltimore, MD, USA.

**Keywords:** sepsis, septic shock, critical care, metabolomics, biomarker

## Abstract

Though there have been developments in clinical care and management, early and accurate diagnosis and risk stratification are still bottlenecks in septic shock patients. Since septic shock is multifactorial with patient-specific underlying co-morbid conditions, early assessment of sepsis becomes challenging due to variable symptoms and clinical manifestations. Moreover, the treatment strategies are traditionally based on their progression and corresponding clinical symptoms, not personalized. The complex pathophysiology assures that a single biomarker cannot identify, stratify, and describe patients affected by septic shock. Traditional biomarkers like CRP, PCT, and cytokines are not sensitive and specific enough to be used entirely for a patient's diagnosis and prognosis.

Thus, the need of the hour is a sensitive and specific biomarker after comprehensive analysis that may facilitate an early diagnosis, prognosis, and drug development. Integration of clinical data with metabolomics would provide means to understand the patient's condition, stratify patients better, and predict the clinical outcome.

## Introduction

Sepsis is a heterogeneous syndrome caused by microbial infection leading to systemic infection development of various complexities spanning from hypotension, organ failure, and shock. Sepsis accounted for 29.5% of patients admitted to the ICU and 18.0% of patients already in the ICU in one of the studies 1. This illustrates the utmost health loss along with a significant economic burden in healthcare, with evidence supporting the continuous increase in the incidence of sepsis and septic shock 2. Sepsis is reported to have a high mortality rate of 40% 3. The study says sepsis is a leading cause of death in intensive care units, with one-third of mortality within the first 48 hours.

The pathophysiology of sepsis has yet to be comprehensively understood, but it is evident that this syndrome involves a complex interplay between the pathogen and host immune system 4. The current treatment strategy for sepsis and septic shock is to maintain homeostasis and prevent multiple organ failure 5. It has been reported that the early diagnosis hours are critical in patient mortality, known as the "golden hour". Early recognition and management reduce the mortality rate by 2% each hour of delay of appropriate antimicrobial therapy 6. There are various therapeutic strategies used by clinicians like immunotherapy 7, hemodynamic optimization 8, 9, and pulmonary-artery catheterization 10, 11. However, none of these strategies gave positive results, suggesting clinicians should examine interventions earlier in the disease. 12.

This led to early goal-directed therapy (EGDT) for treating patients with septic shock 11. EGDT consists of an early hemodynamic assessment based on physical findings and vital signs to detect persistent global tissue hypoxia to rapidly adjust cardiac preload, afterload, and contractility to balance oxygen delivery with oxygen demand. Several studies have been performed to test its efficacy 13, 14. EGDT is still under scanner for general acceptance as a valuable and reproducible treatment strategy. Septic shock mortality is still high due to the lack of predictive parameters for monitoring patients' response and drug delivery.

This paves the way for precision medicine, as clinicians and scientists are becoming aware that to manage these critical illnesses, individuals' response to therapy is essential 15. Precision medicine includes the genome and other omics like metabolome and proteome 16. Metabolomics is particularly interesting because metabolites, the end product of metabolism, result from gene and protein function 17, 18. Thus, it is a better way to understand the phenotype of cells, tissue, organs, or whole organisms. Metabolomics could help identify the molecular pathways obliterated in sepsis and septic shock. The present study's leading idea is to provide insight into the ways involved in septic shock to promote early intervention and personalized treatment.

## Sepsis and Septic Shock

### Definition

The Third International Consensus Definitions for Sepsis and Septic Shock (Sepsis-3) defined sepsis as life-threatening organ dysfunction caused by a dysregulated host response to infection, and septic shock is a subset of sepsis in which underlying circulatory and cellular/metabolic abnormalities are profound enough to increase mortality substantially 19. Patients are identified with septic shock due to persisting hypotension with or without vasopressors to maintain MAP ≥ 65 mm Hg despite adequate fluid resuscitation. There is a critical loss in tissue perfusion, acute failure of multiple organs like lungs, kidneys, and liver, and increased mortality.

### Description: Signs and Symptoms

Sepsis manifests the activation of both the arms of the immune system - pro-inflammatory and anti-inflammatory immune response against the microbe. This, along with activating pattern recognition receptors by monocytes, neutrophils, and macrophages, releases chemokines, cytokines, reactive oxygen species, and proteases. The activation of pattern recognition receptors results in coagulation and complement system activation due to the capillary leak brought about by the loss of endothelium integrity. This chain of events manifests the clinical symptoms of sepsis, which progresses to septic shock 20. Sepsis initiates a systemic inflammatory response and ends with multi-organ failure.

Some of the earlier signs of the systemic inflammatory response are:

a. Fever

b. Tachycardia

c. Tachypnea

d. Leukopenia

This systemic inflammatory response syndrome with an infectious source defines sepsis 21. The development of lactic acidosis is due to the shift from aerobic to anaerobic respiration due to tissue hypoxia brought about by the action of hypotension 21, 22. Some of the early signs of severe sepsis are 23:

a. Hypoxia

b. Hypotension

c. Cyanosis

d. Brain dysfunction

e. Oliguria

f. Ileus

Despite adequate fluid resuscitation, this persistent hypotension results in septic shock 24.

#### Risk factors Description: Signs and Symptoms

Risk factors that are susceptible to sepsis are 23:

a. Diabetes

b. Malignancy

c. Chronic kidney and liver disease

d. Use of corticosteroids

Immunosuppressed state

e. Burns

f. Major surgery

g. Trauma

h. Presence of indwelling catheters

i. Prolonged hospitalization

j. Hemodialysis

k. Extremes of age

### Epidemiology

Male, older age group, and immune-compromised patients are more susceptible to sepsis and septic shock. Sepsis at a younger age is attributed to inheritance 25. Sepsis is the third leading cause of mortality among hospitalized patients worldwide 26, 27. In the United States, there are nearly 850,000 admissions to intensive care for sepsis and septic shock 28. The incidence of sepsis and septic shock increases by 9% annually 29 (Martin et al., 2003). The number of patients admitted with sepsis and septic shock has risen from 600,000 to over 1,000,000 annually from 2000 to 2008 30. Sepsis and septic shock are some of the leading causes of death among hospitalized patients worldwide. Studies have reported a 25% and 50% mortality rate of severe sepsis and septic shock patients, respectively 31. Moreover, the overall mortality rate of sepsis and septic shock is 30% to 50% 32 based on age, gender, race, and organ dysfunction 33. A population-based analysis by Global Burden of Diseases, Injuries, and Risk Factors Study (GBD) 2017 reported 48•9 million cases of sepsis deaths worldwide 5.

Data from India are minimal and usually documented as microbiological profiles and resistance pattern 34. Sepsis-related ICU mortality in India was underestimated by the study conducted by the European Prevalence of Infection in Intensive Care - II (EPIC II) involving 75 countries 35 due to the study being a 1-day point prevalence, which is not all sepsis-related deaths happen in an ICU setting. Information regarding the epidemiology of sepsis in India is in the form of type of infection, not in terms of sepsis 36-38. However, one study considered sepsis as a syndrome and reported that ICU mortality was 56%, hospital mortality was 63.6%, and 28-day mortality was 62.8% in an Indian population cohort 34. The most common causative agent in sepsis was Gram-negative bacteria. This study was a 4-day point prevalence study from 124 ICUs across India, which reported 28.3% of patients with septic shock and had ICU mortality of 18.1% 39. On the other hand, a five-year study from a single center reported the incidence of sepsis as 6%, out of which 16% were hospital-acquired infections 34.

The rising trend of sepsis incidence shows an exponential rise in healthcare expenditure, accounting for 5% of total United States hospital costs 40. With the development of newer sepsis management strategies provided by Surviving sepsis management, there has been a decline in its rate. The mortality rate declined from 16.5% to 13.85% from 2009 to 2012. Only 34% of the survivors have been reported to have recovered from its iatrogenic complications with the consequences of long-term morbidity, quality of life, health issues, and severely impaired outcomes due to associated comorbidities, age, and malignancies 41, 42. Consequently, delays in clinical decision-making, prior diagnosis, and the absence of targeted and tailored therapies can worsen the outcome.

### Diagnosis

Infection anywhere in the body can lead to sepsis and septic shock-like post-surgical infection, urinary tract infection, or pneumonia. There is an absolute need to keep any condition in the body in check to prevent it from spreading and metamorphosing into sepsis or septic shock. There are various screening tools, from a paper checklist to undergoing electronic machine-based diagnosis, whose aim is to detect it as soon as possible, even at the pre-hospital stage 43-46. Early warning signs 47 and the Rothman index 48 can be considered for detection and diagnosis. The Surviving Sepsis Campaign (SSC) website (www. survivingsepsis.org) enlists various screening and diagnosing tools. Conventional clinical parameters are considered as heart rate, respiratory rate, temperature, blood pressure, oxygen saturation, heart rate, respiratory rate, lactate, urea, C- C-reactive protein, blood count, blood cultures, urine cultures, and other bodily fluid cultures to check for sources of infection. Apart from that, imaging techniques are also helpful for confirming condition.

### Pathophysiology

Shock is a continuum of life-threatening events that, when not prevented, will act in concordance and ultimately lead to death. The appropriate treatment would reduce mortality when administered early, but the events are almost irreversible once organ failure is ensured.

When a pathogen enters a host body, it is recognized through pattern recognition receptors (PRRs) like toll-like receptors (TLRs) on the surface of innate immune cells. These PRRs identify two types of molecules: pathogen-associated molecular patterns (PAMPs) and damage-associated molecular patterns (DAMPs). PAMPs are highly conserved structures present on the surface of the invading microbes. DAMPs, on the other hand, are endogenously produced due to cell damage. Activation of PRRs results in the release of inflammatory mediators, local vasodilation, and endothelial permeability, followed by activation of coagulation pathways. This response is hyperinflammatory in sepsis and septic shock and cannot be amended by an anti-inflammatory response. Moreover, excessive reactive oxygen species (ROS) production damages proteins, lipids, and DNA. The coagulation system is also obliterated, causing the formation of thrombi in small blood vessels, causing tissue perfusion 49, 50.

Inflammation and coagulation intertwined in septic shock. Uncontrolled inflammation promotes a syndrome of massive platelet activation, thrombin production, and impaired fibrinolysis called disseminated intravascular coagulopathy (DIC). These two events synergistically result in coagulopathy and bleeding, leading to organ failure. At the systemic level, these events lead to tissue hypoxia due to tissue perfusion, which leads to shock.

The aberration at the cellular level initiates reduced perfusion of the tissues and organs. As a result, the cells and tissues have to shift to anaerobic respiration and metabolism, resulting in increased production of lactate and CO_2_. When not checked, this will result in necrosis and cell death, leading to a decline in tissue functions. Simultaneously, there is a cardiac failure at the systemic level, to which the body compensates by performing peripheral vasoconstriction. This vasoconstriction will shunt the blood circulation to the heart and brain away from the splanchnic artery. Thus, as the shock progresses, it results in renal hypoperfusion, urine output, and anuria. Anoxia and anuria result in acidosis, which supplements heart failure. The body indemnification is insufficient to stabilize the blood volume lost, resulting in hypotension and cardiac damage. Cardiac output loss raises intrapulmonary venous pressure, accumulating blood in pulmonary circulation, resulting in edema and affecting lung functions, paving the way to general hypoxia.

As hypotension continues, blood starts to clot in small vessels; simultaneously, toxins are released from tissues and the intestine, suffering from ischemia. Anoxia results in the release of inflammatory cytokines, which increases the permeability of the vessels, leading to loss of fluids. All these events ultimately result in intense tissue integrity deterioration.

This is the last phase of shock, which is irreversible. It is characterized by a decline in cardiac output, vasodilation of peripheral blood vessels, and eventually death, regardless of the medical interventions. When shock reaches this stage, organs begin to fail, and multi-organ failure (MOF)occurs. This is a condition in which organs not affected directly by trauma or infection become dysfunctional due to hyperimmune response and endothelial dysfunction. The cardiac muscle is damaged to a great extent that even if adequate blood volume is restored and blood pressure is maintained, the patient's heart is inefficient and dysfunctional, ultimately leading to death.

### Management

Major recommendations by the Surviving Sepsis Campaign (SSC) are discussed below:

#### Antimicrobial therapy and source control

Before initiating any antimicrobial treatment, microbiological cultures are performed from bodily fluids (blood, urine, respiratory cultures, and cerebrospinal fluid) and tested for antibiotic resistance. SSC recommends intervening as soon as possible on any site that could be a potential source of infection. For example, any abscess should be drained for an intraabdominal source of infection. It has been reported that the delay in controlling the source would increase the mortality by 6 hours 51 Early goal-directed therapy (EGDT) by Rivers and the group was the first to establish the golden hour in sepsis to decrease mortality. Another study by Kumar et al. demonstrated that antibiotic initiation within the first hour of observed sepsis symptoms would increase survival rate by 79.9%, and every hour delay was associated with a decrease in survival rate by 7.6% 52

SSC recommends an initial broad-spectrum antimicrobial treatment that reaches an adequate concentration in the tissue of the source of sepsis. This should continue for 3-5 days only, and the patient should be brought back to the appropriate single antimicrobial therapy. The treatment should stop based on the low procalcitonin level, which is indicative that the patient is no longer septic.

#### Fluid resuscitation

Studies on EGDT for fluid resuscitation showed mortality benefits 11 (Rivers et al., 2009). However, the results reproducibility was a challenge in other clinical trials, namely ProCESS (a randomized trial of Protocol-based Cre for Early Septic Shock), ProMISE (Protocoloized Managemen in Sepsis), and ARISE (The Australasian Resuscitation In Sepsis Evaluation) 53, 54 Various studies emphasized macrocirculatory resuscitation; however, studies on microcirculatory resuscitation need to be performed.

#### Choice of fluids

There is no ideal fluid that could be used for humans. At present, we have two choices: crystalloids and colloids. The isotonic crystalloid most commonly used is 0.9% sodium chloride. This can cause renal vasoconstriction and mesangial contraction and impair renal function over time 55. The SPLIT trial compared buffered crystalloid solution versus normal saline and observed that buffered crystalloid compared with saline did not reduce AKI risk 56. Comparison between albumin and normal saline in critically ill patients showed some mortality benefits but was not statistically significant 57. However, albumin cannot be used because it is not cost-effective; moreover, there were no mortality benefits in a recent meta-analysis 58, 59.

#### Maintenance of fluids and de‐resuscitation

Fluid resuscitation comprises four stages: rescue, optimization, stabilization, and evacuation or de-resuscitation 60. The rescue improves the perfusion deficits, optimization, and stabilization to maintain the perfusion, while de-resuscitation removes excess fluids to prevent edema. In a study, the mortality of patients treated with restrictive fluid management improved from 33.2% to 24.7% 61.

#### Hemodynamic monitoring

EGDT was based on hemodynamic monitoring of CVP, MAP, and ScvO2. Definite algorithms to guide fluid and pressure therapy exist 62; however, no studies suggest improving septic shock mortality. According to the SSC guideline, repeated focused exams of re-assessment of volume status and tissue perfusion and lactate clearance are critical.

#### Vasoactive agents in the management of septic shock

A major clinical criterion for septic shock is the need for a vasopressor to maintain a MAP of 65 mmHg and serum lactate level >2mmol 19. Vasoactive agents like norepinephrine, dopamine, phenylephrine, epinephrine, vasopressin/terlipressin, and inotropes such as dobutamine and milrinone are commonly used. The first-line drug recommended in septic shock is norepinephrine. Norepinephrine is preferred because it has been reported to reduce mortality and prevent cardiovascular damage 63.

#### Organ support

SSC suggests prone positioning over supine and higher positive end-expiratory pressure (PEEP) over lower PEEP in patients with sepsis-induced acute respiratory distress syndrome. In patients with diabetes as an underlying condition, SSC recommends a protocolized approach to blood glucose management in patients with sepsis, maintaining upper blood glucose levels ≤180 mg/dL. SSC suggests against using intravenous hydrocortisone to treat septic shock patients if adequate fluid resuscitation and vasopressor therapy can restore hemodynamic stability. In cases where it is not achievable, intravenous hydrocortisone at 200 mg per day can be considered in refractory shock 64.In the case of acute kidney injury related to septic shock, the optimal timing of starting renal replacement therapy is still controversial 65, 66.

#### Nutrition

Assessment of nutritional status using scoring systems such as NUTRIC score and NRS 2002 and considering patients' co-morbid conditions is essential. A critically ill patient would need 25-30 kcal/kg on average. Patients with high nutrition risk should be provided with >80% of estimated requirements and a high protein dose. SSC guidelines suggest providing antioxidants and trace minerals but not probiotics 67

## Metabolic changes in sepsis and septic shock

The main goal of human metabolism is to provide energy. Metabolism involves various interconnected pathways. The liver and kidney are the two most active organs involved in metabolism and are highly susceptible to sepsis 68. Thus, tracking any aberrations in the metabolic process would help in an early diagnosis of sepsis. This section deals with the relationship between glucose and fatty acids and changes in amino acids.

### Glucose and fatty acids in sepsis

Under homeostasis or a normal state, glucose and lipids are the primary and secondary energy sources 69. In sepsis, the interdependent relationship between glucose and F.A.s is lost with elevated protein and lipid catabolism compared to normal 69, 70. In sepsis, there is a decline in glucose utilization by extra-hepatic tissues and an increase in the catabolism of adipose tissue and proteins. This would result in hyperglycemia and high amino and free fatty acid levels 71. These events could be attributed to insulin insensitivity due to a decline in glucose uptake and inhibition of insulin on lipid and protein catabolism by the liver.

There is an exponential increase in the rate of VLDL-TAG production due to elevation in the circulatory FFAs and glucose. Simultaneously, an increase in F.A. uptake and storage by fatty tissue as TAGs.It is also common in a non-septic individual to meet an unmet energy requirement, but in sepsis, it occurs in an uncontrolled, compulsive manner.

In the fasted state of sepsis, TAG has enhanced catabolism in muscles and adipose tissues. The FFAs from adipose tissue are exported while other tissues utilize their TAG stores as energy sources 70, resulting in elevated circulatory FFAs.Under normal circumstances, these F.A.s would lead to ketogenesis by the liver and their utilization as an energy source by beta-oxidation. However, these excessive FAS are used in sepsis to synthesize TAGs and secreted as VLDLs 70. The decline in ketogenesis makes the peripheral tissue dependent on proteolysis and gluconeogenesis in a fast state^70^. These mentioned aberrations result in enhanced dependency upon the anaerobic metabolism of glucose. Since the liver and kidney play an essential role in these metabolic alterations, abnormal kidney and liver markers could be utilized in sepsis.

### Amino acids in sepsis

The aberrations in amino acid metabolism due to sepsis are the output of their enhanced utilization as a source of energy and protein catabolism 69. There are two types of amino acids: glucogenic and ketogenic, and some amino acids are both glucogenic and ketogenic.

Metabolism of ketogenic amino acids (lysine and leucine) in sepsis is affected due to the inhibition of ketogenesis and beta-oxidation^71^. Amino acids, which are partially or completely dependent upon the liver for the first stages of catabolism (20 amino acids), would increase rapidly 72 except the aliphatic and branched-chain amino acids which undergo their initial stages of catabolism in skeletal muscles 73. Thus, sepsis-induced liver injury does not cause an increase in BCAA. Certain non-essential amino acids like arginine and tyrosine are produced in the kidney. Acute kidney injury due to sepsis could lead to these amino acids as essential amino acids.

Thus, sepsis is associated with aberration in the metabolism of glucose, amino acids, and fatty acids. This is mainly due to the dysfunction of the liver and kidney, the main organs responsible for metabolism. Thus, these metabolic signatures could differentiate patients with such sepsis-related disorders.

### Clinical challenges: Early diagnosis of sepsis-induced organ dysfunction

There are nine studies of biomarkers for patients with sepsis. Together, they have analyzed the diagnostic value of seven biomarkers: cystatin C (CysC), neutrophil gelatinase-associated lipocalin (NGAL), urinary alpha-glutathione S-transferase (α-GST), tumor necrosis factor-alpha (TNF- α); and serum TNF- α receptors type one and two (sTNF- α - R.I. and II). However, these studies had certain drawbacks: inconsistency in the definition of AKI and evaluation parameters for the diagnostic value of the markers.

Amongst the four of the nine studies mentioned, Neutrophil gelatinase-associated lipocalin was evaluated as a biomarker. Work by Märtensson et al. illustrated the predictive ability of NGAL in urine and serum 74.

The following study compared sepsis with non-infected systemic inflammatory response syndrome (SIRS) 75. Patients with sepsis reported a higher level of NGAL in both plasma and urine compared to SIRS, and the difference in the level of NGAL in urine persisted for 24 hours.

The third study was performed on the serum samples of children with septic shock 76. The fourth one examined the plasma NGAL in the emergency department 77. Both studies gave variable results. The third study showed a low positive predictive value of NGAL, while the third gave a negative predictive value.

Cystatin C was studied in two of the nine studies previously mentioned. The work by Nejat et al. studied the predictive value of Cystatin C in plasma and urine 78. Urinary Cystatin was non-diagnostic at the onset of AKI, and plasma Cystatin was inaccurate at predicting AKI development. Plasma Cystatin was also illustrated as a poor predictor by the works of Mazul-Sunko et al.

The predictive ability of IL-6 has been assessed by two studies 79, 80. The study by Iglesias also evaluated TNF α and TNF- α receptor I and II. Among the four biomarkers studied, only TNF- α receptors I and II were demonstrated to predict septic shock-induced AKI. The last study of this group studied α-GST as a biomarker but was inefficient in predicting sepsis-induced AKI.

Several biomarkers have already been reported as prognostic markers of sepsis, such as C-reactive protein (CRP) 81, lipopolysaccharide-binding protein (LPS) 82 and procalcitonin (PCT) 83. However, these markers must be adequately specific and sensitive to be used as a single biomarker in clinical practice. The impending mortality and morbidity demand symptomatic biomarkers for early diagnosis and prognosis at an early stage. Combining various biomarkers and broad medical consultation, including specialists from different fields, may improve the prognosis and thus reduce overall mortality 84, 85.

## Metabolomics

Clinicians worldwide face challenges for better disease diagnosis and management for targeted therapy. Diseases like septic shock, which are heterogeneous and multifactorial, require a targeted approach; thus, general therapeutic interventions fail to improve mortality. Therefore, there is an urgent need to understand better such multifactorial conditions, which are inflicted with multiple biological insults and etiologies. Many reductionist approaches for discovering biomarkers were performed, but inadequate sensitivity and specificity were reported, thus could not find its clinical application 82, 83. To address this problem, selection methodologies like omics were used to analyze a larger dataset and perform data integration following an interdisciplinary approach 86. Critical illness management and diagnosis are revolutionalized by omics science by aiding in biomarkers identification, drug discovery, and management, paving the way for preventive and personalized medicine 87

Metabolomics is a widespread and rapidly growing omics science, finding various applications in biological sciences like biomarker discovery, drug development and efficacy, and better healthcare management 88. Metabolomics involves the analysis of minor metabolites, which are the end product of cellular metabolism. Thus, metabolomics provides the metabolic fingerprint in real time or at a precise time point. Another essential aspect of metabolomics is understanding the metabolite alteration concerning the effect of environmental factors and underlying pathological state 89.

There are two types of metabolomic analysis: targeted and untargeted. Targeted metabolomics involves analyzing a specific set of metabolites selected based on the biochemical question that needs to be addressed. The drawback of targeted metabolomics is that it requires a prior understanding of the compound of interest. Thus, this method is less acceptable for discovering and identifying metabolic biomarkers 90. Untargeted metabolomics has a universal scope due to the need for prior knowledge of metabolites. This method involves measuring and analyzing all the sets of metabolites present without any bias.

The review focused on providing the comprehensive information on sepsis and its advancement in metabolomics as an approach to improve the diagnosis and prognosis of sepsis and septic shock. Metabolomic is sepsis, and septic shock will provide us with early identification of infection using biomarkers. Metabolomics is a logical approach for biomarker discovery in critical illnesses because the complexity of the disease requires insight into the involved pathological mechanisms. Biomarkers mirror the critical events in the pathogenesis of sepsis and meaningfully reflect the effect of therapies on sepsis development. Analyzing potential biomarkers in serum offers an intriguing way of addressing certain key questions (i)To identify and elucidate disease-associated biomarkers using metabolomics with multivariate statistics and a computational approach. (ii)To predict the mortality outcome by identifying the critical metabolic patterns in survivors and non-survivor groups considering the demographic patterns. (iii)To identify the metabolite fingerprint during the pathogenesis and progression of the disease and identify the key metabolites. (iv)To identify the metabolites that could be used as a parameter for assessing the responsiveness of patients to drug treatment.

There are various studies performed on patients with sepsis and septic shock to deduce biomarkers for sepsis and septic shock.

The first clinical studies for metabolic profiling in critically ill patients were performed in trauma patients 91, 92, as shown in Table 1. They performed an analysis between two groups: uninfected SIRS and multi-organ failure patients, survivors, and non-survivors of septic shock using 1H NMR. Work by Wang et al. was performed using whole blood, wherein the SIRS was correlated to an increase in BCAA and glucose. In contrast, multi-organ failure patients were associated with increased free fatty acids, creatinine, and lactate. The following study by Cohen et al. reported increased lipids and glucose, ketone bodies, and lactate in non-survivors. Both studies above demonstrated non-survivors characterized by an increase in lipids and lactate in the serum sample. Thus supporting the role of lipid and lactate as biomarkers in the clinical outcome of trauma patients and the significance of metabolic profiling in critically ill patients.

Following in lines were studies by Park et al. 93. The analysis compared albumin to placebo in treating acute lung injury (ALI) using serial plasma samples of patients from a random controlled trial. It was reported that there was an improvement in oxygenation in treatment with albumin compared to placebo.

Stringer et al. first published metabolic changes associated with sepsis 94. The study included a univariate analysis of metabolites in patients with ALI and healthy controls using plasma-based 1H NMR metabolomics. They reported a significant increase in adenosine, glutathione, and phosphatidylserine and decreased sphingomyelin in ALI compared to healthy control. These metabolites illustrate aberrations in energy utilization, endothelial barrier with oxidative stress, and apoptosis in septic shock.

The following study by Bruegel et al. in 2012 was whole blood-based metabolomics using LC-MS-MS 95. The study included LPS-activated and non-activated entire blood samples, wherein multivariate analysis was performed to identify nine metabolites of discriminatory potential. These were amino acids, five arachidonic acids, and two cyclooxygenase metabolites.

Non-targeted metabolomics was performed by Liang et al., who used serum samples from healthy controls and septic shock patients 96. The identified biomarkers were validated in the independent patient cohort and are listed as sphingosine, 5-methylcytidine, and 3-dehydrocarnitine. Their study demonstrated that mass spectrometry-based metabolomics can be used for the early diagnosis of septic shock.

A study by Jaurila et al. analyzed septic shock and healthy control by 1H NMR spectroscopy 97. They validated previous findings of septic shock biomarkers. They reported elevated levels of creatinine, 3 hydroxybutyrate, glycoprotein, and glycine and a decline in the concentration of citrate and histidine in septic shock.

Works by Pandey et al. illustrated the metabolic alteration in patients with sepsis and septic shock due to the presence of comorbid conditions like diabetes and hypertension 98, gender 99, and alteration in metabolic profile during disease or progression of the disease 100, 101.

A recent study by Li et al. (2023) identified nine differential metabolites with potential differential diagnosis of sepsis, namely: 3-phenyl lactic acid, N-phenylacetylglutamine, phenylethylamine, traumatin, xanthine, methyl jasmonate, indole, and l-tryptophan 102.

Another study by Chen et al. 103 illustrated seventy-three differentially expressed metabolites (|log2 fold change| > 1.5, adjusted P value < 0.05 and variable importance in the projection (VIP) > 1.5) that could predict sepsis.

Schmerler et al. (2012) demonstrated the difference in metabolic profiles of sepsis from non-infected SIRS. They reported acylcarnitine and glycerophosphatidylcholines as discriminatory markers of sepsis 104.

The next study by Blaise, 2013, performed the NMR-based plasma metabolic analysis of sepsis in trauma patients 105. The study illustrated the elevation of TCA intermediates, BCAA ketone bodies, and allantoin.

Another study by Kamisoglu, Calvano, et al., in 2015, was performed in the Community-Acquired Pneumonia and sepsis Outcome Diagnostics (CAPSOD) cohort between LPS-induced endotoxemia in healthy volunteers and patients with sepsis and non-infected SIRS. This study illustrated that the LPS-induced endotoxemia and sepsis groups had 16 significant metabolites. It was reported that there was a decrease in lysophosphatidylcholine upon comparing these groups with non-infected SIRS. In addition, acylcarnitine was found to be a discriminatory biomarker for mortality in sepsis.

The following milestone study by Langley in 2013 examined the metabolic alterations in the CAPSOD cohort and analyzed the 28-day survivors and non-survivors 85. The study's primary objective was to differentiate survivors and non-survivors of sepsis. They reported a list of metabolites, including 12 amino acids, 11 nucleic acids, TCA cycle intermediates, and free fatty acids.

Using a similar cohort from CAPSOD, Seymour et al. performed another analysis 106. This study included patients with sepsis from Genetic and Inflammatory Markers of Sepsis (GenIMS), which were analyzed using LC-MS-based metabolomics. Statistical analysis was performed to distinguish sepsis survivors and non-survivors (with 90-day survival) to identify metabolites of oxidative stress, bile acid, nucleic acid, and stress. Amongst all, pseudouridine was highly significant. These discriminatory metabolites were explored for further examination in animal models of sepsis and reported increased pseudouridine levels in the mice model's liver and kidney.

Another study in 2016 compared the metabolic profiles based on the type of infection 107. They illustrated that certain specific metabolites could be used for distinguishing the different types of infections. Glycerophospholipid was found to be clear with CAP in sepsis and septic shock.Similarly, the decline in acetylornithine would be reflective of BSI.They demonstrated that putrescine, lysoPCaC18:0, and SM C16:1 were related to non-survivors in sepsis with community-acquired pneumonia, intra-abdominal infections, and bloodstream infections, respectively.

A recent study by Feng et al. 108 identified acrylic acid, 5-amino-3-oxohexanoate, 3b-hydroxy-5-cholenoic acid, cytidine, succinic acid semialdehyde, PE [P-18:1(9Z)/16:1(9Z)], sphinganine, uracil, and uridine as a potential biomarker of sepsis and septic shock. One of the studies employs metabolic analysis of sepsis and septic shock in a pediatric population, wherein serum samples are collected from septic shock, SIRS, and healthy control 109. The group performed a non-targeted metabolomics approach to identify 2 hydroxybutyrate, lactate, histidine, phenylalanine, and arginine as discriminatory metabolites for septic shock from non-infected SIRS and healthy control. They even demonstrated a mortality model for detecting metabolites that could be used as a marker for mortality.

The third study was performed between patients with sepsis, non-SIRS, and healthy control using LC MS-based metabolomics; they also categorized sepsis as uncomplicated sepsis and severe sepsis or septic shock 110. Analyzing the group with non-SIRS and sepsis demonstrated a statistical decrease in lactitol dehydrate and S-phenyl-d-cysteine and an increase in S-(3-methylbutanoyl)-dihydrolipoamide-E and N-non-anoyl glycine in sepsis compared to the non-infected SIRS.

Kauppi, Alicia Edin, et al., 2016, performed LCMS-based metabolomics of whole blood from patients with bacteremic sepsis and ER controls 111. Using the regression model, they identified 107 metabolites and validated them, reducing the number of metabolites stepwise until the final 6 significant metabolites were obtained.

Another work by Mickiewicz in 2014 demonstrated that 1H NMR could be used as a diagnostic tool for septic shock 112, 113. They performed metabolomics analysis between septic shock and ICU controls. A reduction in BCAAs and urea cycle-related amino acids, including glutamine, glutamate, and arginine, was revealed in septic shock patients. On the other hand, aromatic amino acids (AAAs) and proline were increased.

Numerous studies are being designed to provide the biomarkers of mortality in sepsis and septic shock for better management of critical illnesses. One such study demonstrated the application of LC-MS in differentiating survivors and non-survivors of septic shock 114. They reported increased metabolites linked to the TCA cycle, amino acids, and ketone bodies in non-survivors and decreased urea cycle metabolites and FA metabolism metabolites. They demonstrated that six metabolites could be used as an early predictor of mortality in septic shock.

Ferrario et al. examined septic shock patients by categorizing them into 28-day and 90-day mortality groups 115. Spermidine, putrescine, and glucogenic amino acids are elevated in non-survivors of septic shock. In non-survivors, an increase in kynurenine concentration was observed from day 1 to day 7, whereas a decrease in phosphatidylcholines and lysophosphatidylcholines was found to be significant.

Another targeted metabolomics study was performed for lipid profiling and its association with mortality in septic shock 116. The study demonstrated a significant elevation in prostaglandin F2a and leukotriene B4 in non-survivors, whereas elevated resolvin E1, resolvin D5, and 17R-protectin were reported in septic shock survivors.

A study explored the association of acetylcarnitine with sepsis and its mortality 117. They demonstrated that non-survivors had higher levels of plasma acetylcarnitine when compared to survivors and that it could be used to predict 28-day mortality risk.

A study by Rogers et al. showed that, in two separate cohorts, the individual metabolites and their network were associated with 28-day mortality.They identified gamma-glutamyl phenylalanine, gamma-glutamyl tyrosine, 1-arachidonoylGPC(20:4), taurochenodeoxycholate, 3-(4-hydroxyphenyl) lactate, sucrose, kynurenine)^118^ . Another work by Chung et al. confirmed that plasma acetylcarnitine could be used as a prognostic marker with the potential to mirror organ dysfunction, inflammation, and infection in sepsis 119.

One of the latest works on septic shock mortality was performed by Liu et al. to reveal the metabolic profile of septic shock survivors and non-survivors using 0hr and 24 hr serum samples 120. The study focused on providing the metabolic evolution from 0 to 24 hours in the serum of septic shock survivors and non-survivors.

Wang et al. performed a meta-analysis that included 1287 individuals in 21 cohortswith 2509 metabolite comparisons. The identified included amino acids, mitochondrial metabolism, eicosanoids, and lysophospholipids. 121.

Another unique study by Garcia Simon's group performed a metabolic analysis of septic shock using urine samples collected from septic shock patients 122. They performed an analysis between survivors and non-survivors at 0 hours and the first 24 hours of admission to identify the metabolic signatures of mortality in urine samples. Using the urine metabolomics approach, they identified arginine, methionine, phenylalanine hippurate, and ethanol as markers of mortality.

Another work by Huang et al. 123 illustrated a risk classification of phenylalanine- and leucine in patients with sepsis and septic shock. While Winkler et al. 124 stated that Symmetrical (SDMA) and asymmetrical dimethylarginine (ADMA) in sepsis are combined high-risk markers for sepsis survival.

Alice et al. 125 studies revealed a dynamic change in metabolite levels over the study period in severe septic shock patients stratified for mortality. Meanwhile, Evans et al. 126 demonstrated a decreased phenylalanine in septic shock non-survivors at one year.

There are two studies reported to monitor the treatment response in sepsis 127, 128. Using a metabolomics study, the first study aimed to identify patients who would reduce vasopressor use after L-carnitine supplementation 127. Patients with a good response had a low level of carnitine and acetylcarnitine, where methionine, lysine, phenylalanine, and tyrosine were found to be increased after L-carnitine was given 127. The second study categorized septic shock patients based upon the sequential organ failure assessment (SOFA) score as responders and non-responders 128. An untargeted metabolomics comparing the degree of metabolite changes between the responders and non-responders over (from baseline to 48 h) found that the concentration of myristic acid and oleic acid were illustrated comparatively more decline, whereas creatinine showed a comparatively less decline in the responders than the non-responders. When comparing the degree of metabolite changes over time, kynurenine was increased in the responders but lower than in the non-responders. Most SMs, SM(OH)s, and PCs were increased in the responders, whereas they were decreased in the non-responders, these phospholipids had a predictive ability to determine treatment response.

Another study by Pandey et al. 2023 illustrates the use of metabolomics to understand the responsiveness of patients to drugs administrated during their prolonged hospital stay in patients with sepsis and septic shock.^129^. These findings suggest that metabolomics can be used to monitor treatment response in sepsis effective in patient management.

## Conclusion

Though metabolomics has been available for more than a decade, there has been continuous development and advancement in the field, continuously assisting in enhancing the feasibility and accessibility of the analysis with low economic cost. Since the past couple of years, scientists' interest in metabolomics has increased because it analyzes a set of common molecular weight molecules downstream of the genome and proteome. Since metabolites are very sensitive to genetic, physiological, or environmental stimuli, they have found application in various fields. This attribute of metabolomics is advantageous for analyzing complex conditions like sepsis and septic shock, making way for personalized medicine.

Personalized medicine is designing treatment strategies, considering the individual specific underlying conditions and clinical symptoms and their genetics, environment, and lifestyles 130. Indeed, metabolomics assessment of sepsis and septic shock is critical because it could be helpful for physicians in septic shock management and pave the way for personalized therapy. Moreover, we could help understand the complex mechanisms currently still under study involved in the pathogenesis and progression of septic shock.

## Figures and Tables

**Figure 1:**
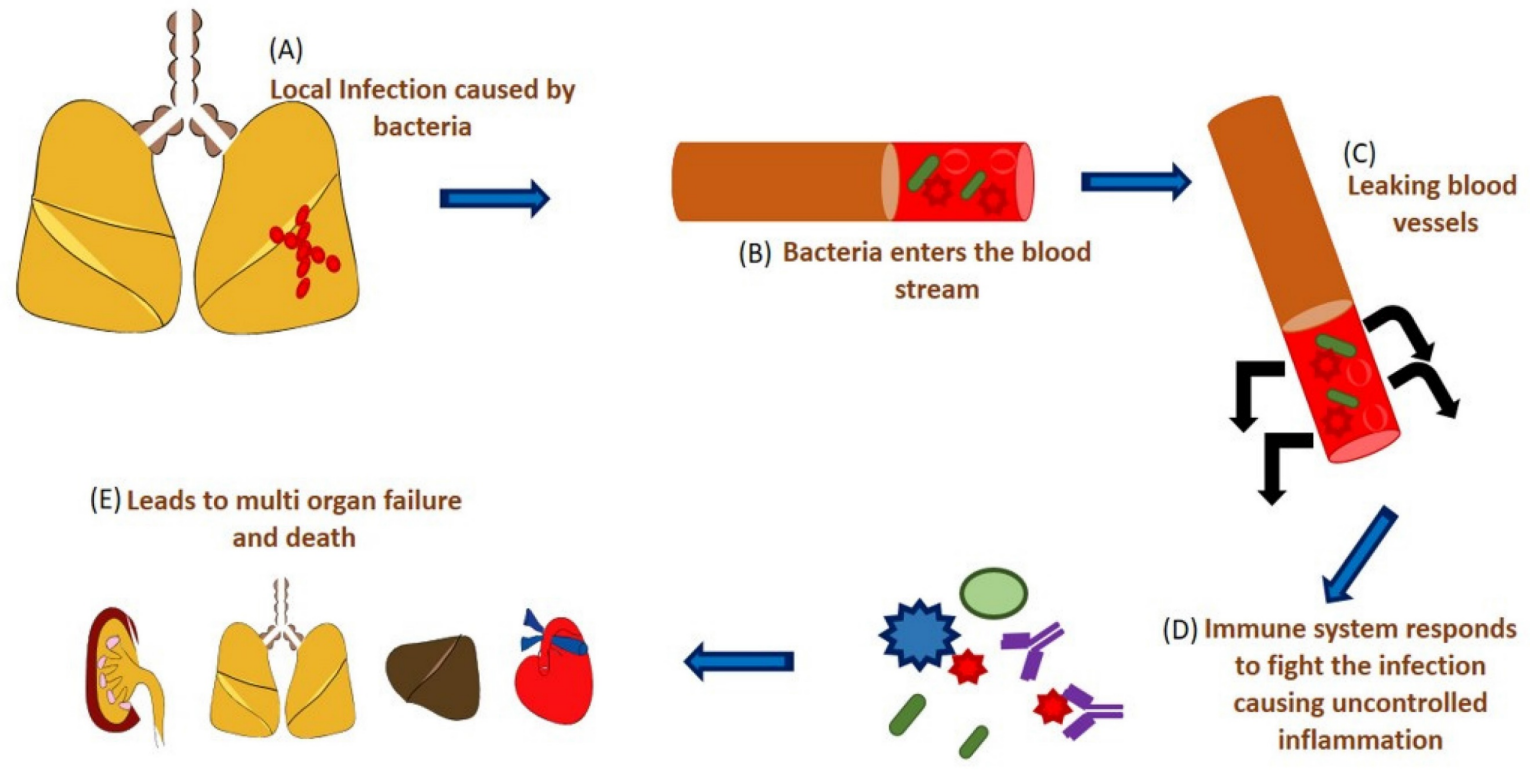
Schematic representation of sepsis and septic shock. (A) A localized infection caused by microbes, (B) the microbes enter the system, (C) leaking of the blood vessels, (D) immune response by the body causing uncontrolled hyperinflammation, (E) which starts affecting various organs causing multi-organ failure.

**Figure 2:**
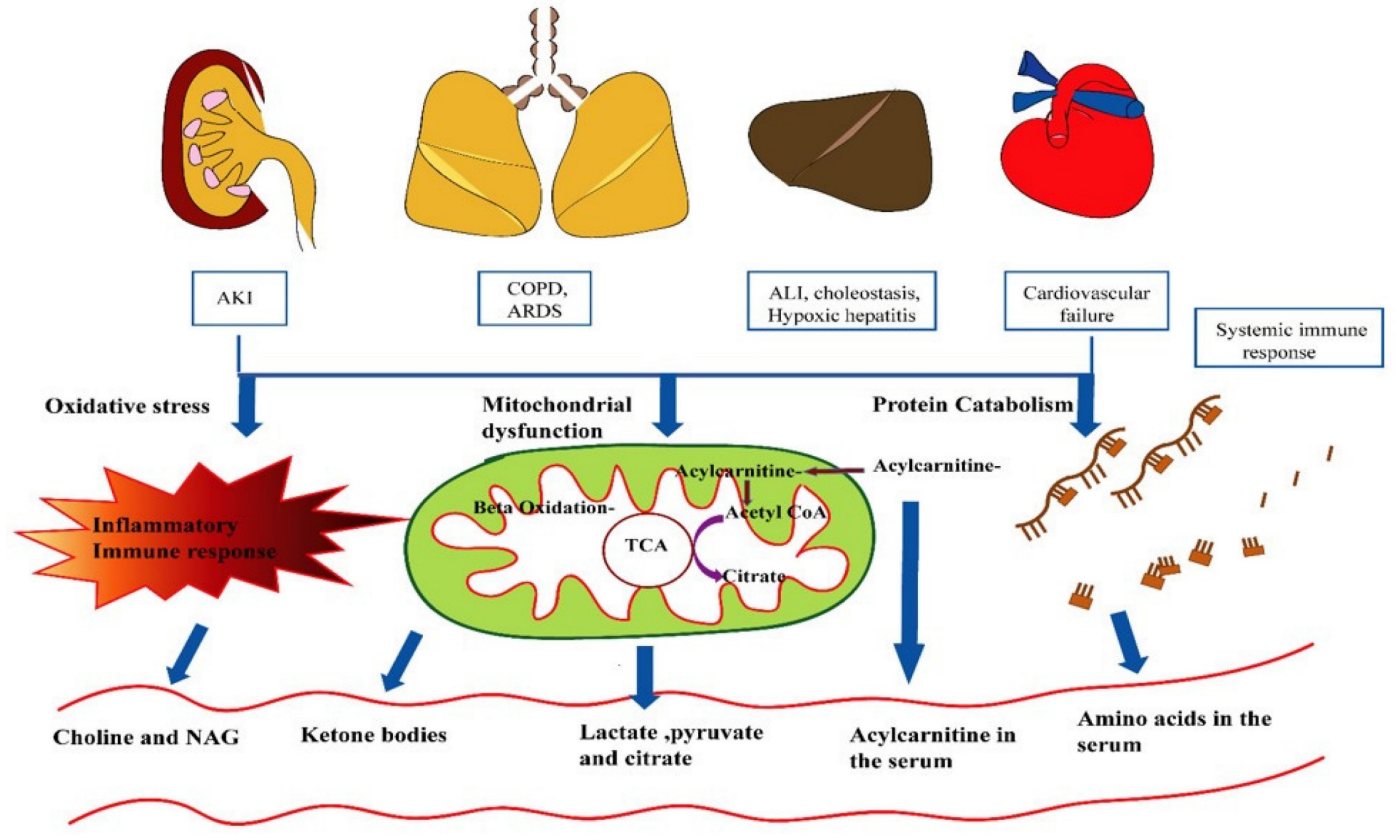
Schematic representation of pathways obliterated in septic shock. Highlighted in red are metabolites altered in septic shock. Briefly, sepsis induces acute kidney injury (AKI) in the kidney, leading to hypoxia in the lungs and liver. This is followed by COPD (Chronic obstructive pulmonary disease) and ARDS (acute respiratory distress syndrome) in the lungs, cardiovascular failure, and acute liver injury.

**Figure 3:**
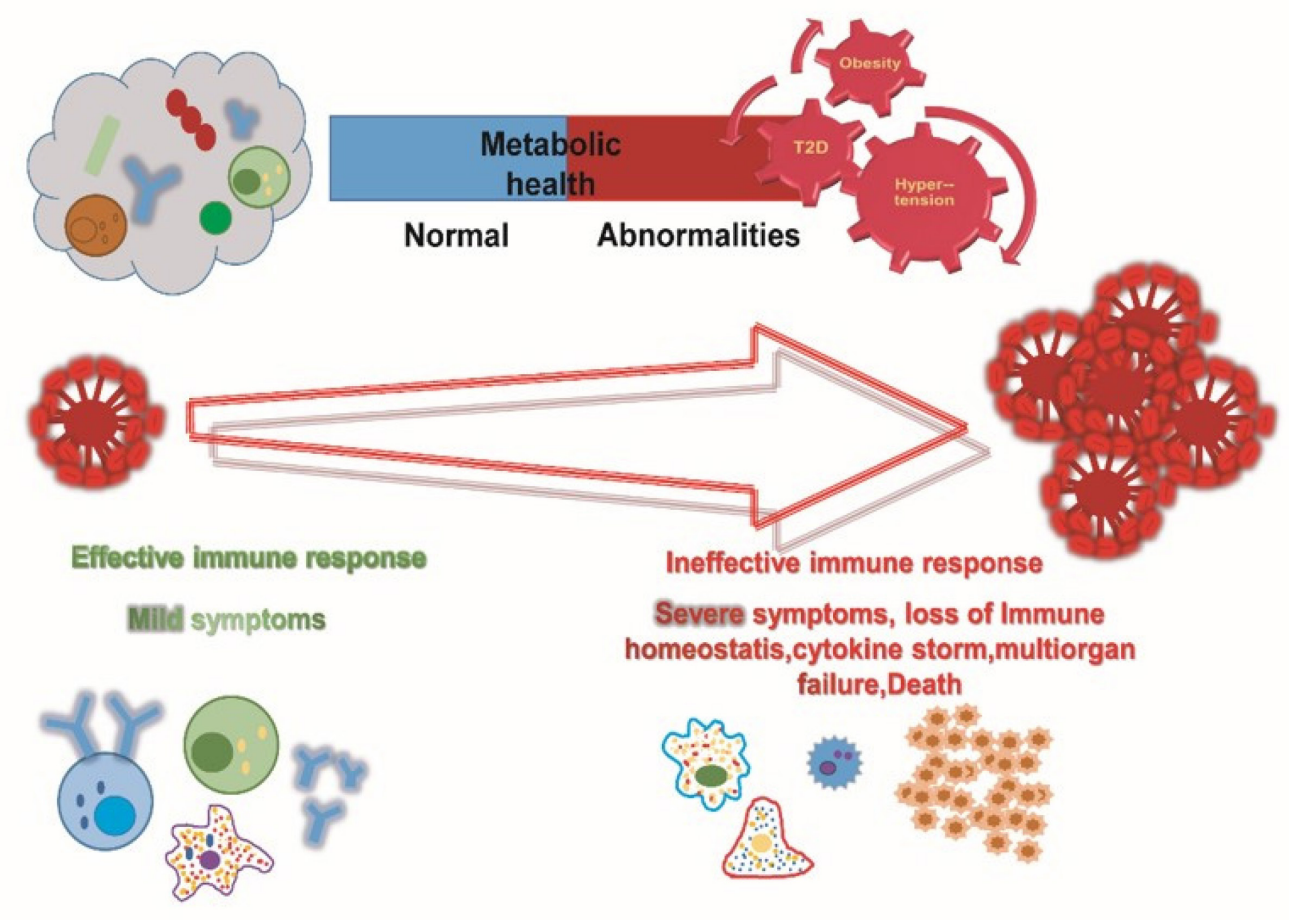
Schematic representation of metabolic changes in response to immune response.

**Table 1:** Clinical metabolic profiling studies of sepsis and septic shock.

Authors	Year	Platform used	Subjects	Metabolite identified
Mao et al	2009	^1^H NMR	a. Severe trauma and SIRS b. Severe trauma and MODs c. Healthy controls	MODS vs SIRS and both trauma vs healthy controls: MODS: ↑ free fatty acids, glycerol, creatinine and lactate; SIRS:↑ amino acids (predominantly BCAAs) and glucose.
Cohen et al	2010	^1^H NMR	a. Severe trauma survivors b. Severe trauma and non-survivors c. Healthy controls	Survivor vs non-survivors and both trauma vs healthy control: ↑glucose, glutamate, ketone bodies, lactate,TAGs, mono-unsaturated fatty acids and glycerophospholipids.
Park et al	2011	^1^H NMR	a. Acute lung injury treatment b. Acute lung injury placebo c. Healthy control	Acute lung injury treatment vs acute lung injury placebo: ↑lysyl moiety of albumin; alanine, LDL, VLDL, valine, cholesterol.
Stringer et al	2011	^1^H NMR	a. Sepsis related ALI b. Healthy controls	Sepsis related ALI vs healthy control ↑ adenosine, glutathione and phosphatidylserine; ↓sphingomyelin
Bruegel et al	2012	LC-MS/MS	a. Sepsis b. Healthy controls	LPS activated whole blood vs healthy controls: ↓ in AA, PGE2, 11-HETE; TXB2
Schmerier et al	2012	LC-MS/MS	a. Sepsis b. Non-infected SIRS	Sepsis vs non infected SIRS: ↑acyl-carnitines and glycerophosphatidylcholines: and a diacyl-glycerophosphatidylcholine
Langley et al	2013	LC-MS/MS	a. Sepsis non-survivors b. Sepsis survivors c. Non-infected SIRS	(1) Sepsis non-survivors vs survivors: ↑ 17 amino acid catabolites, 16 carnitine esters, 11 nucleic acid catabolites, citrate, dihydroxyacetone, malate, pyruvate, four free fatty acids; in addtion to ↓ seven GPCs and GPE, ↑ lactate, and acyl-carnitines (2) Sepsis survivors vs non-infected SIRS: ↓ citrate and malate, glycerol, glycerol 3-phosphate, phosphate, 21 amino acids and their catabolites, 12 GPCs and GPE esters, and six carnitine esters
Blaise et al	2013	^1^H NMR	a. Trauma + sepsis b. Trauma - sepsis	Trauma + sepsis vs trauma - sepsis: ↑ aspartate, citrate, valine, hydroxybutyrate and allantoin
Seymour et al	2014	LC-MS/MS		Sepsis non survivors vs survivors: ↑ taurochenolate sulfate and glycochenolate sulfate; ↑ cortisol, cortisone, and sulfated hormones allantoin, N1-methyladenosine, N2, N2-dimethylguanosine, N6-carbamoylthreonyladenosine and pseudouridine
Su et al	2014	LC-MS/MS	a. Severe sepsis b. Uncomplicated sepsis c. Non-infected SIRS d. Healthy controls	(1) Sepsis vs non infected SIRS: ↓ lactitol dehydrate and S-phenyl-d-cysteine and ↑ in S-(3-methylbutanoyl)-dihydrolipoamide-E and N-non-anoyl glycine. (2) Severe sepsis vs uncomplicated sepsis: ↓ glyceryl-phosphoryl-ethanolamine, Ne, Ne-dimethyl-lysine, phenylacetamide and d cysteine (3) death within 24 hours: ↓ S-(3-methylbutanoyl)-dihydrolipoamide-E, phosphatidylglycerol, glycerophosphocholine and S-succinyl glutathione
Kamisoglu et al	2015	LC-MS/MS	a. Sepsis non-survivors b. Sepsis survivors c. LPS induced endotoxemia in healthy control d. Non infected SIRS	(1) Sepsis and healthy control vs LPS induced endotoxemia vs non infected SIRS: ↑ 2-hydroxybutyrate, mannose, bilirubin and lipids (2) Sepsis survivors vs non-survivors: ↑ acyl-carnitines were the most discriminatory metabolites
Mickiewicz et al	2013	^1^H NMR	a. Septic shock b. SIRS c. Healthy control	Discriminatory metabolite between septic shock, SIRS and healthy control: 2 hydroxybutyrate, lactate, histidine, phenylalanine and arginine
Mickiewicz et al	2014	^1^H NMR	a. Septic shock b. ICU controls	Discriminatory metabolite between survivors and non survivors of sepsis 20 metabolites differentiated the profiles of survivors and non survivors
Garcia-Simon et al	2015	^1^H NMR	a. Septic shock	Discriminatory metabolite between survivors and non survivors of sepsis Arginine, methionine, glutamine, phenylalanine, glucose, ethanol, and hippurate showing differences between non-survivor/survivor
Liu et al	2016	LC-MS/MS	a. Septic shock	Discriminatory metabolite: 43 significant metabolites varied in their levels when compared between survivors with non-survivors. Six primary discriminators: Valine, leucine, isoleucine, citrulline, carnitine 2:0, and betanine
Ferrario et al	2016	LC-MS/MS	a. Septic shock	Upregulated in non-survivors: Polyamines, glucogenic amino acids, and kynurenine Downregulated in non-survivors: phosphatidylcholines and lysophosphatidylcholines.
Neugebauer et al.	2016	LC-MS/MS	a SIRS b Sepsis	Discriminatory metabolite: acylcarnitines, glycerophospholipids and sphingolipids were altered in sepsis compared to systemic inflammatory response syndrome.
Cambiaghi et al	2018	LC-MS/MS	a. Septic shock	Discriminatory metabolite: Alteration in the lipidome of non- survivors was found. PC aa C42:6, PC aa C40:6, and lysoPC species
Dalli et al	2017	LC-MS/MS	a. Septic shock	Discriminatory metabolite: Elevation in the levels Prostaglandin F2α, leukotriene B4, resolvin E1 resolvin D5, and 17R-protectin D1 were found in non-survivors.
Chung et al	2019	UHPLC-MS	a. Septic shock	Discriminatory metabolite: A significantly higher level of plasma acetylcarnitine was found in sepsis non-survivors when compared with survivors.
Liu et al	2019	^1^H NMR	a. Septic shock	Discriminatory metabolite: The concentrations of alanine, glutamate, glutamine, methionine, aromatic amino acids, ketone bodies, 3-hydroxybutyrate, and acetate were increased in the non-survivors as compared to the survivors. N-acetyl glycoprotein level was found decreased in non-survivors.
Jaurila et al	2020	^1^H NMR	a. Sepsis b. Healthy control	Discriminatory metabolite: Significantly higher serum lactate and citrate concentrations in non-survivors compared with survivors.
Reisinger et al.	2021	^1^H NMR	a. Sepsis b. Healthy control	Discriminatory metabolite: BCAA (valine, leucine, isoleucine, 3-hydroxybutyrate) was significantly lower in sepsis non-survivors.
Liang Q et al.	2016	LC-MS/MS	Septic shock b Healthy control	Discriminatory metabolite: Sphingosine, 5 methylcytidine, 3 dehydrocarnitine, 4 acetamido-2-aminobutanoic acid and phenyllactic acid in the Septic shock subjects were significantly different from the controls.
Feng et al.	2022	LC-MS/MS	a Multiple trauma (non SIRS) b Sepsis	Discriminatory metabolite: Nine potential biomarkers, namely, acrylic acid, 5-amino-3-oxohexanoate, 3b-hydroxy-5-cholenoic acid, cytidine, succinic acid semialdehyde, PE [P-18:1(9Z)/16:1(9Z)], sphinganine, uracil, and uridine were identified
Mecatti et al.	2018	LC-MS/MS	a Septic shock b Healthy control	Discriminatory metabolite: Fatty acids and phospholipids detected in plasma and erythrocytes could signal sepsis vs. non-sepsis. Lyso-PCs and SMs were downregulated, whereas the saturated and unsaturated phosphatidylcholines (PCs) were upregulated in the plasma and erythrocytes of septic patients.
Pandey et al.	2020	^1^H NMR	a Septic Shock b Healthy control Septic shock with co morbid conditions (diabetes and hypertension) Septic shock with primary diagnosis (respiratory illness and encephalopathy)	Discriminatory metabolite: The potential biomarkers for septic shock are lactate, 3 hydroxybutyrate, 3 hydroxyisovalerate, proline, 1,2 propanediol, creatine, glycine, phenylalanine, and myoinositol, bile, NAG, and VLDL, which were significantly upregulated in septic shock patients,w hereas citrate, carnitine, HDL, LDL, and lipoprotein with phosphocholine head group were downregulated in septic shock patients.
Pandey et al.	2023	^1^HNMR	a Septic shock pre treatment b Septic shock post treatment	Discriminatory metabolite: The study showed time-dependent metabolite alteration in ketone bodies, amino acids, choline, and NAG in patients undergoing treatment.
Cambiaghi A et al.	2017	LC- MS/MS	Treatment response in Septic shock	Discriminatory metabolite: lipidome alterations play an important role in individual patients' responses to infection. Furthermore, alanine indicates a possible alteration in the glucose-alanine cycle in the liver, providing a different picture of liver functionality from bilirubin.
Kauppi et al.	2016	LC-MS/MS	a Sepsis b Healthy control	Discriminatory metabolite: Six metabolites were identified for bacteremic sepsis.
Cambiaghi A et al.	2018	LC - MS/MS	a Septic shock	Discriminatory metabolite: Identified circulating lipids and coagulation cascade in septic shock progression
Winkler et al.	2018	LC- MS/MS	Sepsis	Discriminatory metabolite: SDMS and ADMA associated with sepsis mortality
Huang et al.	2019	LC- MS/MS	Septic shock	Discriminatory metabolite: Phenylalanine- and leucine-defined risk classifications provide metabolic information with prognostic value for patients with severe infection.
Li et al.	2023	LC-MS/MS	a Sepsis b Healthy control	Discriminatory metabolite: 3-phenyl lactic acid, N- Discriminatory metabolite: phenylacetylglutamine, phenylethylamine, traumatin, xanthine, methyl jasmonate, indole, l-tryptophan
Cheng et al.	2022	LC-MS/MS	a Sepsis b Healthy control	Discriminatory metabolite: Seventy-three differentially expressed metabolites that could predict sepsis were identified.
Pandey et al.	2022	^1^H NMR	a Septic shock survivor (M/F) b Septic shock non survivor (M/F)	Discriminatory metabolite: The energy-related metabolites, ketone bodies, choline, and NAG were found to be primarily responsible for differentiating survivors and non-survivors. The gender-based mortality stratification identified a female-specific association of the anti-inflammatory response, innate immune response, and β oxidation, and a male-specific association of the pro-inflammatory response to septic shock
Puskarich MA	2015	LC- MS/MS	Carnitine treatment response in Septic shock	Drug responsive metabolite: Responsive towards carnitine treatment
Evans et al.	2019	LC- MS/MS	Carnitine treatment response in Septic shock	Drug responsive metabolite: metabolic signature of L-carnitine-treated non-survivors is associated with a severity of illness (e.g., vascular inflammation) that is not routinely clinically detected.
Pandey et al.	2023	^1^HNMR	Treatment response in Septic shock	Drug responsive metabolite: 3 hydroxybutyrate, lactate, and phenylalanine which were lower, whereas glutamate and choline higher in patients showing responsiveness.

KEY: 11-HETE: 11-hydroxyeicosatetraenoic acid, AA: arachidonic acid, BCAA: branched-chain amino acid, FA: fatty acid, GPC: glycerophosphocholine, MODS: multi-organ dysfunction syndrome, PGE2: prostaglandin E2, SIRS: systemic inflammatory response syndrome, TAG: triacylglyceride, NAG: N acetylglycoprotein, TXB2: thromboxane B2, LDL: low density lipoprotein, VLDL: very low density lipoprotein, ALI: acute liver injury, GPE: glycerophosphoethanolamine, LPS: lipopolysaccharide, UHPLC MS: Ultra-high performance liquid chromatography coupled with mass spectroscometry, M/F: Male/Female
